# The Heart Block Hat-Trick: A Case of Alternating First-, Second-, and Third-Degree Heart Blocks

**DOI:** 10.1155/2023/8664315

**Published:** 2023-06-14

**Authors:** Nicholas Huerta, Salman Malik, Christopher Haas

**Affiliations:** ^1^MedStar Health Internal Medicine Residency Program, Baltimore, MD, USA; ^2^MedStar Health, Department of Cardiology, MedStar Harbor Hospital, Baltimore MD, USA

## Abstract

Idiopathic third-degree atrioventricular (AV) block in a relatively young patient is an uncommon phenomenon. Even more rare is when the third-degree heart block is alternating with the first- and second-degree AV blocks. In this case, we present a 39-year-old man with varying degrees of AV block, alternating the third-degree, second-degree, and first-degree AV blocks. The patient underwent an extensive workup for underlying etiologies, and results were inconclusive. A pacemaker was implanted and set for physiologic pacing via left bundle branch area pacing (LBBAP). This case will discuss potential genetic abnormalities associated with AV block and highlight LBBAP as an emerging technique for physiologic pacing.

## 1. Introduction

The first-degree atrioventricular (AV) block is a relatively common occurrence with a prevalence of 6.84% [1]. This type of heart block is a rather benign condition, and typically, follow-up is not warranted. The second-degree AV block (including Mobitz I and II) is less common with a prevalence of 0.18% [1]. The type of second-degree AV block will dictate the possible interventions. The third-degree AV block is much less common with a prevalence of 0.04% [1], and this type of AV block requires more urgent intervention with identification and treatment of the potential precipitating factor and/or pacemaker implantation. The risk of AV block increases with age and disease burden [1, 2]. It is not uncommon for the second-degree Mobitz II AV block to progress to complete heart block. However, unlike this case, typically, once the disease progresses, the type of AV block will not alternate. Other causes of complete heart block include cardiac ischemia, fibrosis, infiltrative processes (i.e., sarcoidosis, lymphoma, and amyloidosis), inflammatory processes (i.e., lupus, Lyme carditis, and rheumatic fever), medication toxicity, and electrolyte abnormalities.

This is a novel case because the patient was alternating between the full spectrum of heart blocks (third-, second-, and first-degree). This type of alternating heart block is not previously reported in the literature. What makes this case even more uncommon is that the patient was relatively young, and there was no clear precipitant for his diagnosis, leading to suspicion of an underlying genetic abnormality. This case also highlights the novel method of left bundle branch area pacing (LBBAP) as a method to achieve physiologic pacing.

## 2. Case Presentation

A 39-year-old male presented to the emergency department with complaints of intermittent dizziness. He was in usual health until he developed severe, intermittent dizziness three days prior to presentation while celebrating a bachelor party. He denied additional neurologic symptoms, such as loss of consciousness, numbness, tingling, slurred speech, visual disturbances, and unilateral weakness. He also noted pressure-like abdominal discomfort, which accompanied the first episode of dizziness. While the abdominal discomfort self-resolved, the patient's dizziness persisted, worsening with exertion and mildly abating with rest. There were several occasions where he almost lost consciousness, prompting him to sit and rest until the feeling subsided. He had no prior experience with such symptoms. Intriguingly, the patient incidentally noted a heart rate that nadired to 29 beats per minute (BPM) on his smartwatch, which coincided with his symptomatology. He was an active drinker, consuming 3–4 standard drinks daily (including both liquor and beer), with increased consumption to more than 8 drinks per day during the two days he was celebrating at the bachelor party. He denied other recreational drug use. Medical history was unremarkable except for a diagnosis of asymptomatic first-degree AV block, which was diagnosed as part of a routine sport physical during his teenage years. He was never advised to follow-up and never experienced limitations in functional activity. He denied any family history of cardiac diseases. He was noted to have significant longitudinal outdoor exposure on the eastern coast of the United States and had multiple episodes of childhood tick bites, without a prior diagnosis of Lyme disease. There were no known recent known tick bites, but he removed a tick from his daughter's scalp one week before admission. He denied recent illness and other symptoms, such as vomiting, diarrhea, constipation, and rash. He was not taking any medications aside from the occasional nonsteroidal anti-inflammatory medication for musculoskeletal pain.

**Figure 1:**
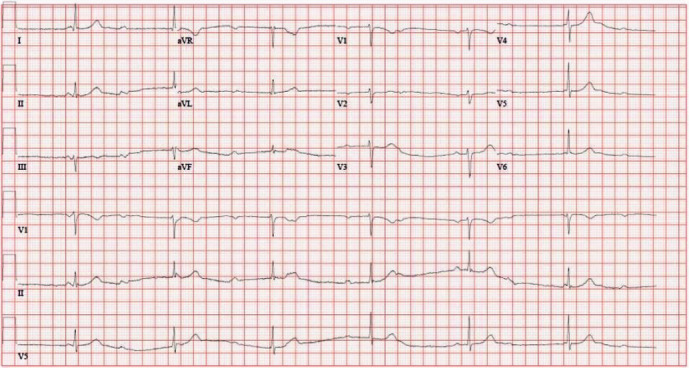
Presenting ECG, which demonstrates third-degree AV block with a narrow complex ventricular escape rhythm of 36 BPM.

**Figure 2:**
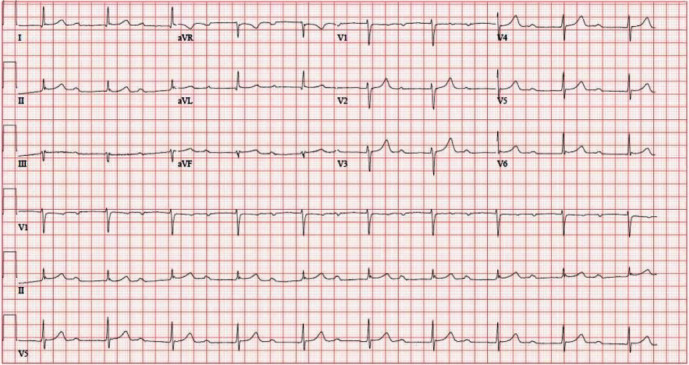
Follow-up ECG, which demonstrates first-degree AV block with markedly prolonged PR interval of 512 ms.

**Figure 3:**
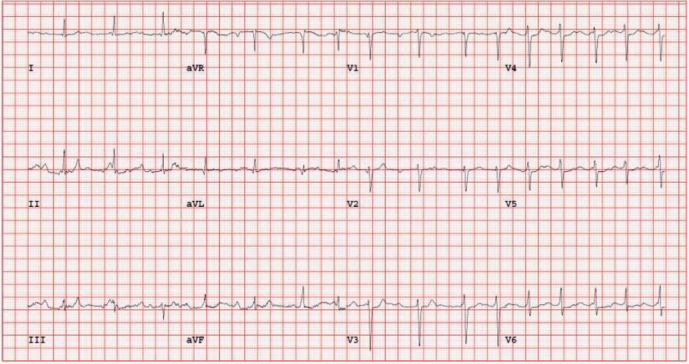
ECG stress test at peak exercise, which demonstrates intermittent high-grade 2 : 1 AV block.

Several electrocardiograms (ECGs) performed at the time of presentation in the emergency department demonstrated third-degree AV block (Figure 1). Patient was immediately placed on continuous cardiac telemetry, and after one hour, his heart rate improved and appeared to have transitioned to first-degree AV block on the monitor. The transition to first-degree AV block was later confirmed on an ECG performed about 10 hours after arrival (Figure 2). Subsequently, during an exercise ECG stress test, the patient was found to have high-grade intermittent 2 : 1 second-degree AV block at rest and with exertion (Figure 3). The patient's basic metabolic panel, all electrolytes (including potassium, magnesium, and calcium), and complete blood count with differential were completely normal. Other laboratory diagnostics, including high sensitivity troponin, anti-nuclear antibody, serum and urine protein electrophoresis, human immunodeficiency virus antibody-antigen, corona virus polymerase charin reaction, and Lyme disease serologies were unremarkable. The only abnormal laboratory findings were an elevated high-sensitivity C-reactive protein (hsCRP) to 10.03 mg/L (reference 0–3 mg/L) and mildly elevated alanine transaminase (ALT) to 73 U/L (reference 10–49 U/L). Transthoracic echocardiogram showed borderline concentric left ventricular hypertrophy and an enlarged right ventricle but was otherwise normal. The pulmonary artery pressure could not be assessed due to inadequate Doppler signal. To evaluate for infiltrative disease, cardiac magnetic resonance imaging was performed and was completely unremarkable.

## 3. Management

In the emergency department external pacemaker pads were placed and atropine was ordered to the bedside for rapid administration in the setting of unstable or symptomatic bradycardia. The patient remained hemodynamically stable and relatively asymptomatic while resting in the hospital bed and did not require the atropine. An infectious disease specialist evaluated the patient and began empiric treatment with doxycycline given concern for Lyme carditis. The patient was subsequently transferred to a tertiary care hospital. Lyme titers resulted negative, but the patient insisted that he remains on antibiotics. While the infectious disease team was reluctant to continue antibiotics, they acquiesced to the patient's desire to remain on antibiotic therapy. ECG stress test was performed to assess for chronotropic competence and further characterize his AV nodal disease [3]. The findings of a high-grade 2 : 1 heart block with exercise prompted intervention with pacemaker implantation. He underwent successful dual chamber pacemaker implantation using LBBAP in order to establish more physiologic conduction.

Device interrogation in the clinic two weeks after pacemaker implantation demonstrated normal sinus rhythm at a rate of 46 BPM. The device had been programmed conservatively (AAI <> DDD 40–170) to favor native conduction. Interestingly, device interrogation demonstrated atrial pacing 9% of the time and ventricular pacing only 0.1% of the time. The atrial pacing primarily occurred at lower heart rates, suggesting sinus node dysfunction. The AV block had resolved on follow-up ECG. He was re-tested for Lyme disease, and the results remained negative. The patient returned to work and did not experience further episodes of dizziness. It was recommended for the patient to proceed with genetic testing for the possibility of a familial conduction abnormality.

## 4. Discussion

There was no clearly identifiable cause for this patient's complete heart block. The geography and history were concerning for Lyme carditis. However, infectious, structural, infiltrative, and ischemic causes were all ruled out. Alcohol-mediated myocardial toxicity was possible given the elevated ALT and hsCRP [4], but felt to be unlikely because the patient had not consumed alcohol in several days and prior reports of acute alcohol-induced AV block demonstrated resolution of conduction abnormalities upon sobriety [5]. It is possible that the non-specific findings of borderline enlarged right ventricle with left ventricular hypertrophy were an early sign of alcohol-related cardiomyopathy.

Interestingly, the patient had several ECGs, which demonstrated multiple conduction abnormalities: first-degree, second-degree (Mobitz II), and third-degree AV blocks. When coupled with the negative diagnostic workup and reported history of first-degree AV block as a teenager, this raised concern for an underlying genetic abnormality affecting the conduction system. A possible culprit is the *SCN5A* gene. This gene encodes the cardiac voltage-gated sodium channel *α*-subunit, and mutations are associated with several conduction abnormalities, including AV block [6, 7]. The specific mutations associated with AV block are G298S and D1595N. The mechanism is through a reduction in the density of sodium currents via a decrease in fast-inactivation, delayed recovery from fast inactivation, and an increase in slow inactivation of the sodium channels [7]. A mutation in the *LMNA* gene is also possible given its association with AV block in younger patients [8]. A newer disease entity known as idiopathic paroxysmal AV block (IPAVB) was also considered. It is theorized that this condition is associated with hyper-affinity of adenosine receptors localized in the AV node [9]. However, our patient failed to satisfy diagnostic criteria for IPAVB as it requires the absence of other rhythm disturbances prior to experiencing complete heart block; his history of first-degree AV block precluded this diagnosis. Given there was no identification of the precipitating factor for his heart block, it was recommended that he undergo genetic testing during outpatient follow-up.

**Figure 4:**
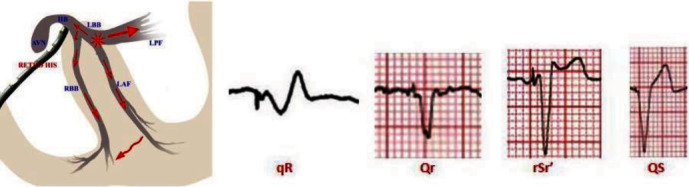
Vectors of depolarization with potential ECG findings in patients with LBBAP (courtesy of Das et al. [14]). The initial *q*/*Q* wave is due to the leftward and posterior direction of the initial depolarization. The RBBB pattern is due to this delayed depolarization of the right side. The QRS is narrow because of a normal functioning right bundle branch resulting in rapid conduction to the right ventricle in addition to the slower transseptal depolarization wave.

LBBAP was used as an alternative to right ventricular pacing and His bundle pacing (HBP). LBBAP may avoid the common complication of ventricular dyssynchrony and pacemaker induced cardiomyopathy (PiCM), which can result from right ventricular pacing. The estimated prevalence of PiCM is 12% and on average takes 4.3 years to develop [10, 11]. The more traditional method for physiologic pacing is HBP, but LBBAP is an emerging technique which overcomes some of the limitations of HBP. According to some experts, LBBAP is the best way to achieve physiologic pacing as it avoids the complications and procedural difficulty inherent to HBP [12, 13]. The limitations of HBP include high anatomical variability, small size, higher pacing output (because of the surrounding fibrous body) leading to premature battery depletion, high capture thresholds leading to increased rates of septal pacing, and lead revisions [14–16]. Common ECG findings with LBBAP include narrow QRS complex with right bundle branch block (RBBB) pattern in V1 (Figure 4). The RBBB pattern is from two wavefronts of depolarization. One wavefront traverses the physiologic anterograde pathway down to the Purkinje fibers, whereas the other wavefront travels retrograde to the His bundle and then down the right bundle. However, the anatomic variability and large left posterior fascicle can lead to essentially two different areas of pacemaker capture with LBBAP: the main left bundle area or the left posterior fascicle. Pacing at the left posterior fascicle will result in left axis deviation due to a left anterior fascicular delay, whereas pacing at the main left bundle area will result in normal axis [13]. The two most common complications of LBBAP are septal perforation and thromboembolism. While studies on this technique have been promising, there is a paucity of evidence on long-term outcomes and identification of specific patient populations who may benefit most from this type of pacing [17].

## 5. Conclusions

This patient demonstrated significant conduction abnormalities in both the sinoatrial and AV nodes suggestive of a possible genetic abnormality. Symptomatic third-degree AV block requires urgent treatment; however, it is important to rule out reversible causes before proceeding with pacemaker implantation. Some of the commonly recognized reversible causes to be aware of include electrolyte abnormalities, hypothermia, thyroid dysfunction, medication toxicity (i.e., beta-blockers), myocardial ischemia (typically inferiorly), and infectious diseases, such as Lyme disease. In patients with a clear indication for cardiac pacing, LBBAP should be considered and actively investigated as an alternative to HBP for physiologic pacing.

## Data Availability

Data supporting this research article are available from the corresponding author or first author on reasonable request.

## Abbreviations

ECG: Electrocardiogram
BPM: Beats per minutes
AV: Atrioventricular
LBBAP: Left bundle branch area pacing
HBP: His bundle pacing.

## Additional Points

*Learning Objectives.* The learning objectives are as follows.

1. Identify potential etiologies of complete heart block.
2. Understand the role of LBBAP as a newer entity for physiologic pacing.
